# Supplementary material for: Content-rich biological network constructed by mining PubMed abstracts
Source: BMC Bioinformatics. 2004 Oct 8;5:147. doi: 10.1186/1471-2105-5-147 (PMC528731; doi:10.1186/1471-2105-5-147)
Supplement: Additional File 2 — The original results of the above study (non-essential files are deleted to keep the file size under the limit set by BMC bioinformatics). [file 1471-2105-5-147-S2.bz2 › chilibotAdditionalFile2/dip20/173ID9388212E588/html/IL6ST_PTPN11.html]

 


 **IL6ST** and **PTPN11** 
  
Found 54 abstracts in PubMed, retrieved 20.  
 

 What does Google say? 
 PDF only 
| .edu only 

---

**Interactive relationship** (e.g. stimulation, inhibition, etc)

**Inhibitory relationship**- SHP2  [ **PTPN11** ]  and SOCS3 contribute to Tyr 759 dependent attenuation of interleukin 6 signaling through gp130  [ **IL6ST** ] .  Ref: 12403768 J Biol Chem, 2003
- Tyrosine 759 of gp130  [ **IL6ST** ]  is required for the negative regulation of LIF mediated signaling by both the protein tyrosine phosphatase SHP 2  [ **PTPN11** ]  and the suppressor of cytokine signaling 3 SOCS 3.  Ref: 12425940 Brain Res Mol Brain Res, 2002
- Mitogenic hyperresponsiveness of synovial cells to the LIF IL 6 family of cyto kines was caused by sustained gp130  [ **IL6ST** ]  mediated SHP 2  [ **PTPN11** ]  ras Erk activation due to impaired STAT mediated induction of suppressor of cytokine signaling SOCS proteins which normally limits gp130  [ **IL6ST** ]  signaling.  Ref: 11457894 J Exp Med, 2001
- The receptor subunit gp130  [ **IL6ST** ]  transduces multiple cell type specific activities of the leukemia inhibitory factor LIF interleukin IL 6 family of cytokines through the signal transducer and activator of transcription STAT and src homology 2 domain bearing protein tyrosine phosphatase SHP 2  [ **PTPN11** ]  ras Erk pathways.  Ref: 11457894 J Exp Med, 2001
**Neutral relationship**- Reciprocal regulation of gastrointestinal homeostasis by SHP2  [ **PTPN11** ]  and STAT mediated trefoil gene activation in gp130  [ **IL6ST** ]  mutant mice.  Ref: 12219085 Nat Med, 2002
- Immediately after receptor complex activation, signal transducers and activators of transcription STATs 1 and 3 and the Src homology domain containing protein tyrosine phosphatase 2 SHP2  [ **PTPN11** ]  are recruited to gp130  [ **IL6ST** ]  and subsequently tyrosine phosphorylated.  Ref: 11737194 Eur J Biochem, 2001
- We show that receptor and membrane targeted SHP2  [ **PTPN11** ]  counteracts IL 6 signaling independent of SOCS3 binding to gp130  [ **IL6ST** ] .  Ref: 12403768 J Biol Chem, 2003
- Taken together, these findings indicate that Gab1 SHP2  [ **PTPN11** ]  interaction plays a crucial role in gp130  [ **IL6ST** ]  dependent longitudinal elongation of cardiomyoctes through activation of ERK5.  Ref: 12855672 Circ Res< MedlineTA>Circ Res, 2003
- Intriguingly, recent in vivo studies indicated that the SHP 2  [ **PTPN11** ]  binding site and YXXQ mediated pathways through gp130  [ **IL6ST** ]  are not mutually exclusive but affect each other a mutation at the SHP 2  [ **PTPN11** ]  binding site prolongs STAT3 activation, and a loss of STAT activation by gp130  [ **IL6ST** ]  truncation leads to sustained SHP 2  [ **PTPN11** ]  ERK MAPK phosphorylation.  Ref: 12687404 Rev Physiol Biochem Pharmacol< MedlineTA>Rev Physiol Biochem Pharmacol, 2003
- gp130  [ **IL6ST** ]  F759 F759 knockin mice carry a point mutation at the SHP2  [ **PTPN11** ]  binding site of gp130  [ **IL6ST** ]  due to the replacement of tyrosine 759 Y759 for human gp130  [ **IL6ST** ]  with phenylalanine F.  Ref: 12070777 Genes Immun, 2002
- These data suggest, that there are two, largely distinct modes of negative regulation of gp130  [ **IL6ST** ]  activity, despite the fact that both SOCS3 and SHP2  [ **PTPN11** ]  are recruited to the same site within gp130  [ **IL6ST** ] .  Ref: 12403768 J Biol Chem, 2003
- We generated a mouse line in which the src homology 2 domain bearing protein tyrosine phosphatase SHP 2  [ **PTPN11** ]  binding site of gp130  [ **IL6ST** ] , tyrosine 759, was mutated to phenylalanine gp130  [ **IL6ST** ]  F759 F759.  Ref: 12370259 J Exp Med, 2002
- Therefore, the joint pathology in gp130  [ **IL6ST** ]  DeltaSTAT mice is likely to arise from the disturbance of the otherwise balanced activation of the SHP 2  [ **PTPN11** ]  ras Erk and STAT signaling cascades emanating from gp130  [ **IL6ST** ] .  Ref: 11457894 J Exp Med, 2001
- Activation of gp130  [ **IL6ST** ]  Transduces Hypertrophic Signal Through Interaction of Scaffolding Docking Protein Gab1 With Tyrosine Phosphatase SHP2  [ **PTPN11** ]  in Cardiomyocytes.  Ref: 12855672 Circ Res< MedlineTA>Circ Res, 2001
- These data suggest that desensitization of vascular cells to OnM is mediated in trans and involves Tyr residue 759 in gp130  [ **IL6ST** ]  but is not mediated by either SOCS 3 or SHP 2  [ **PTPN11** ] , the only two proteins currently known to bind to gp130  [ **IL6ST** ]  at this site.  Ref: 12724316 J Biol Chem< MedlineTA>J Biol Chem, 2001
- We conclude that normal Shp 2  [ **PTPN11** ]  function is critical for the initial step of ES cell differentiation to mesoderm and to hemangioblasts and acts within the LIF gp130  [ **IL6ST** ]  Stat3 pathway to maintain a proper balance of ES cell differentiation, pluripotency, and apoptosis.  Ref: 12791646 Blood< MedlineTA>Blood, 2001
- This indicates that STAT signaling is necessary for LIF and CNTF stimulated VIP and ChAT expression and Y759 of gp130  [ **IL6ST** ]  mediates the activities of SHP 2  [ **PTPN11** ]  and SOCS 3, which act to negatively regulate STAT activity.  Ref: 12425940 Brain Res Mol Brain Res, 2002

**Non-interactive relationship** (e.g. studied together, co-existance, homology, etc.)

- Suppressor of cytokine signaling 3 SOCS 3 and the protein tyrosine phosphatase SHP 2  [ **PTPN11** ]  both regulate signaling by cytokines of the interleukin 6 family, and this is dependent upon recruitment to tyrosine 757 in the shared cytokine receptor subunit gp130  [ **IL6ST** ] .  Ref: 12119038 Biochemistry, 2002
- Cells transfected with a chimaeric receptor containing the SHP2  [ **PTPN11** ]  selective gp130  [ **IL6ST** ]  intracellular domain showed an enhanced response to cytokine stimulation, which was similar to that shown by a chimaeric gp130  [ **IL6ST** ]  receptor mutant carrying a Y757F point mutation that failed to recruit either SOCS3 or SHP2  [ **PTPN11** ] .  Ref: 12593670 Biochem J, 2003
- Furthermore, activation of p38 mitogen activated protein kinase is shown to be essential for the inhibitory effect of TNF alpha on IL 6 signaling and TNF alpha dependent recruitment of SHP2  [ **PTPN11** ]  to gp130  [ **IL6ST** ] .  Ref: 12817006 J Immunol, 2003
- Synovial cells from this mouse exhibited mitogenic hyper responsiveness to cytokines of the LIF IL 6 family, a phenomenon that was caused by sustained gp130  [ **IL6ST** ]  mediated SHP 2  [ **PTPN11** ]  Ras Erk activation due to a defect in the induction of SOCS 1 suppressor of cytokine signaling 1.  Ref: 12010564 Arthritis Res, 2002
- Interestingly, gp130  [ **IL6ST** ]  associated SHP 2  [ **PTPN11** ]  phosphorylation was detected in the IL 6 induced CESS cells but not myeloma cell lines.  Ref: 12040451 Leukemia, 2002
- Here, we evaluated the functions of the two major signaling pathways, the signal transducers and activators of transcription 1 and 3 STAT1 3 and the Src homology tyrosine phosphatase 2 SHP2  [ **PTPN11** ]  Ras ERK, emanating from the common signal transducer, gp130  [ **IL6ST** ] , in the gastrointestinal tract.  Ref: 12219085 Nat Med, 2002
- However, the extent to which hematopoiesis is dependent upon activation of a particular gp130  [ **IL6ST** ]  signaling pathway, namely STAT1 3 or SHP2  [ **PTPN11** ]  MAPK, is unknown.  Ref: 12423677 Exp Hematol, 2002
- ... the Y759 SHP 2  [ **PTPN11** ]  mediated cascade by gp130  [ **IL6ST** ]  stimulation has growth enhancing effects.  Ref: 12687404 Rev Physiol Biochem Pharmacol< MedlineTA>Rev Physiol Biochem Pharmacol, 2002
- On the other hand, SOCS3 inhibits signaling in cells expressing a truncated SHP2  [ **PTPN11** ]  protein, which is not recruited to gp130  [ **IL6ST** ] .  Ref: 12403768 J Biol Chem, 2003
- In the present study, we show the design of a mutant gp130  [ **IL6ST** ]  receptor that can recruit SHP2  [ **PTPN11** ] , but not SOCS3.  Ref: 12593670 Biochem J, 2003
- Gp130  [ **IL6ST** ]  757F mice, with a knock in mutation abrogating SHP2  [ **PTPN11** ]  Ras ERK signaling, developed gastric adenomas by three months of age.  Ref: 12219085 Nat Med, 2002
- In this study we demonstrate that stimulation of macrophages and fibroblast cell lines with TNF alpha causes the recruitment of SHP2  [ **PTPN11** ]  to the gp130  [ **IL6ST** ]  signal transducing subunit and leads to tyrosine phosphorylation of SHP2  [ **PTPN11** ]  and gp130  [ **IL6ST** ] .  Ref: 12817006 J Immunol, 2003
- M Ernst and colleagues generated and studied knock in mice gp130DeltaSTAT DeltaSTAT, in which all STAT binding sites sites binding signal transducers and activators of transcription were deleted from their gene encoding gp130  [ **IL6ST** ]  but binding sites for both Janus kinases JAKs and for the protein tyrosine phosphatase 2 SHP 2  [ **PTPN11** ]  were preserved.  Ref: 12010564 Arthritis Res, 2002
- Negative regulation of gp130  [ **IL6ST** ]  signalling mediated through tyrosine 757 is not dependent on the recruitment of SHP2  [ **PTPN11** ] .  Ref: 12593670 Biochem J, 2003
- To determine whether duration is functionally significant, we transduced EC with a chimeric receptor containing extracellular domains of platelet derived growth factor receptor beta and intracellular regions of gp130  [ **IL6ST** ] , the signaling subunit of the OnM receptor, mutated to prevent binding of the tyrosine phosphatase SHP 2  [ **PTPN11** ] .  Ref: 11777927 J Biol Chem, 2002
- The subsequent recruitment of the common signal transducer gp130  [ **IL6ST** ]  and activation of the JAK STAT and SHP 2  [ **PTPN11** ]  Ras mitogen activated protein kinase MAPK pathways are responsible for the majority of cellular responses elicited by IL 6 cytokines.  Ref: 12764151 J Biol Chem< MedlineTA>J Biol Chem, 2002
- In this context the cytoplasmic SHP2  [ **PTPN11** ]  SOCS3 recruitment site of gp130  [ **IL6ST** ]  tyrosine 759 is shown to be important for the inhibitory effects of TNF alpha, since mutation of this residue completely restores IL 6 stimulated activation of STAT3 and, consequently, of a STAT3 dependent promoter.  Ref: 12817006 J Immunol, 2003
- Since recruitment of the tyrosine phosphatase Src homology protein tyrosine phosphatase 2 SHP2  [ **PTPN11** ]  to the signal transducing receptor subunit gp130  [ **IL6ST** ]  attenuates IL 6 mediated STAT activation, we were interested in whether TNF alpha also induces the association of SHP2  [ **PTPN11** ]  to the gp130  [ **IL6ST** ]  receptor subunit.  Ref: 12817006 J Immunol, 2003
- In this respect murine fibroblasts lacking exon 3 of SHP2  [ **PTPN11** ]  are not sensitive to TNF alpha, indicating that functional SHP2  [ **PTPN11** ]  and its recruitment to gp130  [ **IL6ST** ]  are key events in inhibition of IL 6 dependent STAT activation by TNF alpha.  Ref: 12817006 J Immunol, 2003
- These results demonstrate that the recruitment of SHP2  [ **PTPN11** ]  alone is not sufficient for Y757 dependent negative regulation of gp130  [ **IL6ST** ]  signalling.  Ref: 12593670 Biochem J, 2003
